# A single-center, randomized, open-label, two-period, crossover, fasted bioequivalence study: comparing recombinant human follicle-stimulating hormone for injection (JZB30) with Gonal-f® in healthy Chinese female participants

**DOI:** 10.3389/fphar.2025.1678830

**Published:** 2025-11-10

**Authors:** Zhuo Chen, Qin Yu, Shiyin Feng, Li Mo, Linrui Cai, Chunfeng Du, Dan Du, Qin Zou

**Affiliations:** 1 National Drug Clinical Trial Institution of West China Second University Hospital, Sichuan University, Chengdu, China; 2 NMPA Key Laboratory for Technical Research on Drug Products In Vitro and In Vivo Correlation, Chengdu, China; 3 Key Laboratory of Birth Defects and Related Diseases of Women and Children, Sichuan University, Ministry of Education, Chengdu, China; 4 Chengdu Jingze Biopharmaceutical Co., Ltd., Chengdu Cross-Strait Science and Technology Industrial Development Park, Chengdu, China

**Keywords:** recombinant human follicle-stimulating hormone, pharmacokinetics, bioequivalence, safety, biosimilar

## Abstract

**Objective:**

This study aimed to evaluate the pharmacokinetic (PK) bioequivalence, safety, and immunogenicity of recombinant human follicle-stimulating hormone (rhFSH) for injection (code name: JZB30), developed by Chengdu Jingze Biopharmaceutical Co., Ltd., in comparison with Gonal-f® developed by Merck Serono in healthy adult Chinese female participants.

**Methods:**

single-center, randomized, open-label, two-period crossover study for bioequivalence assessment enrolled 48 healthy adult female participants, who were equally and randomly assigned to two treatment sequences. Each participant received a single subcutaneous injection of either JZB30 or Gonal-f® at a dose of 225 IU on Day 1 and again on Day 11 as part of the crossover design. Blood concentrations of FSH were measured using a validated electrochemiluminescence immunoassay, followed by non-compartmental PK analysis. Safety and immunogenicity were systematically monitored throughout the study period, including adverse events and anti-FSH antibody testing.

**Results:**

The geometric mean ratios (GMRs) of the primary PK parameters—Cmax, AUC_0-t_, and ${AUC}_{0-\infty }$ –after baseline correction were 106.12%, 108.04%, and 115.32%, respectively. The 90% confidence intervals for these parameters (Cmax:101.92%–110.49%; AUC_0-t_:104.13%–112.09%; ${AUC}_{0-\infty }$ ,: 106.47%–124.90%) all fell within the predefined bioequivalence range of 80%–125%. All adverse events (AEs) were mild (Grade 1) and resolved spontaneously without requiring medical intervention. One participant tested positive for anti-FSH antibodies following administration of the reference formulation; however, the antibody response reverted to negative without any intervention, indicating a low risk of immunogenicity.

**Conclusion:**

JZB30 was demonstrated to be pharmacokinetically bioequivalent to Gonal-f® in healthy adult Chinese female participants, with comparable safety profiles and a low risk of immunogenicity. These findings provide evidence to support the clinical application of JZB30 as a biosimilar to Gonal-f®.

**Clinical Trial Registration:**

The trial was registered on ClinicalTrials.gov under identifier NCT06778304.

## 1 Introduction

Assisted reproductive technology (ART) represents a critical strategy for managing female infertility, with controlled ovarian stimulation serving as a central component. Follicle-stimulating hormone (FSH) plays an essential role in this process by pharmacologically inducing the development and maturation of multiple ovarian follicles. FSH, a glycoprotein hormone secreted by the anterior pituitary gland, acts synergistically with luteinizing hormone (LH) to promote oocyte maturation in females. FSH is critical for follicular growth, which provides the structural and functional environment for oocyte development and maturation. It is also essential for triggering ovulation, steroid hormone synthesis in females, and spermatogenesis in males.Gonal-f®, a recombinant human follicle-stimulating hormone (rhFSH) injection developed by Merck Serono, has been widely used in ART since its approval in the United States in 1998, owing to its high purity and stability. JZB30, developed by Chengdu Jingze Biopharmaceutical Co., Ltd., shares the same active ingredient, formulation, specifications, indications, administration route, and dosage regimen as Gonal-f®. This study aims to evaluate the pharmacokinetics, safety, and immunogenicity of JZB30 compared with Gonal-f® in healthy adult Chinese female participants through a single-center, randomized, open-label, two-period, crossover bioequivalence trial, thereby providing evidence to support its rational clinical application.

## 2 Methods

### 2.1 Participants

This study was conducted at the Clinical Research Center of West China Second University Hospital of Sichuan University, Chengdu, Sichuan Province, China, from January 2021 to January 2022. All participants provided written informed consent. The main inclusion criteria were: healthy female participants aged 18–45 years (inclusive), body weight ≥45 kg, and body mass index (BMI) between 18 and 28 kg/m^2^;having a regular menstrual cycle length of 25–34 days (inclusive); sexually active but not seeking pregnancy; normal or clinically insignificant abnormalities in medical history, physical examination, laboratory tests, and gynecological examinations (including uterus and ovaries); normal sex hormone levels or deemed clinically insignificant by the investigator; voluntary participation in the clinical trial, understanding of the study procedures, and signed informed consent. The main exclusion criteria included: polycystic ovary syndrome; a history of ovarian hyperstimulation syndrome (OHSS); allergy to FSH or known allergy to gonadotropin-releasing hormone agonists (GnRH-a) or their analogs; clinically significant abnormalities in the history of ovarian, breast, uterine, hypothalamic, or pituitary diseases; history or current thromboembolic disease; use of hormonal contraceptives (oral short-acting contraceptives within 3 months or long-acting contraceptives within 6 months before screening); drug abuse; blood donation or loss of ≥400 mL within 3 months prior to screening.

### 2.2 Study design

This study was approved by the Ethics Committee of West China Second University Hospital of Sichuan University (approval number Y2020035). The study adhered to the Declaration of Helsinki (World Medical Association, 2013), the principles of Good Clinical Practice (National Medical Products Administration and National Health Commission of the People’s Republic of China, 2020), and all relevant Chinese laws and regulations.

Based on the bioequivalence studies of Gonal-f® biosimilars Ovaleap® (XM17) (Lammerich et al., 2015) and Bemfola® (Wolzt et al., 2016) in Europe, and the clinical use of Gonal-f® in China, the study design followed the European Medicines Agency (EMA) guidelines (European Medicines Agency EMA, 2013) for rhFSH biosimilar and the Chinese Center for Drug Evaluation (CDE) guideline (National Medical Products Administration NMPA, 2015). This Phase I study was a single-center, randomized, open-label, two-period, crossover, single-dose trial assessing the bioequivalence of two rhFSH formulations. A 225 IU (16.5 μg) subcutaneous injection was administered in the umbilical region (3–10 cm from the navel).

We planned to enroll 48 healthy adult female participants. Screening was conducted over 21 days. Eligible participants entered the downregulation phase. On Day −14 (days 15–21 of the menstrual cycle), participants received a single intramuscular injection of triptorelin acetate for injection (Decapeptyl®) 3.75 mg. This was performed to achieve pituitary downregulation, suppressing the secretion of endogenous FSH and thus minimizing its interference on the PK assessment of the exogenous rhFSH formulations. On Day 0, PK blood samples and serum FSH levels were collected (if the downregulation requirement was not met, retesting could be extended up to 4 days). If the participant met the downregulation requirement (FSH ≤4 IU/L) (Lammerich et al., 2015), the inclusion/exclusion criteria were re-evaluated. Those who still met the criteria were randomly assigned to Group A (T-R group) or Group B (R-T group) and underwent baseline assessments. If the participant did not meet the downregulation requirement, she was excluded from the study.

On Day 1, participants received a single subcutaneous injection of the test drug or reference drug (injection site: 3–10 cm around the umbilicus) at a dose of 225 IU. Participants were required to fast for 10 h before and 2 h after each dose in both periods. After an 8–10-day washout period, the second period of crossover dosing began on Day 11. Blood samples for PK analysis were collected at specified time points (see Section 2.3 Sample collection and analysis).

Clinical safety assessments were conducted throughout the trial. Vital signs were monitored daily from the screening period to Day 18 and at the end of the trial. Physical examinations and 12-lead electrocardiograms were performed at screening, Day 0, Day 18, and at the end of the trial (cover both the scheduled end-of-study visit and unscheduled follow-up visits triggered by special events, such as a positive antibody result). Routine blood tests, urinalysis, biochemical tests, and coagulation function tests were conducted at screening, Day 18, and at the end of the trial. Venous blood samples (3 mL) for anti-FSH antibody testing were collected at screening, Day 0, Day 8, Day 18, and at the end of the trial. Adverse events (AEs) were monitored and recorded throughout the trial, with follow-up conducted until resolution of any AE. The detailed procedure is shown in Table 1.

**TABLE 1:** Trial flow.

Trial content	Screening period	Downregulation	Baseline period	First cycle					Second cycle					Follow-up period	Withdrawal from trial
	21 days before downregulation	Menstrual day 15–21	D0	Hospital observation period			Washout period (daily hospital visits)		Hospital observation period			Daily hospital visits		Until menstrual recovery	
		D-14		D1	D2	D3	D4 -D7	D8 -D10	D11	D12	D13	D14-D17	D18		
Screening and Exclusion Criteria Evaluation	**╳**		**╳**												
DafiLin® Administration 1		**╳**													
downregulation evaluation 2								**╳**							
FSH administration 3				**╳**					**╳**						
PK Blood Sample Collection 4			**╳**	**╳**	**╳**	**╳**	**╳**	**╳**	**╳**	**╳**	**╳**	**╳**	**╳**		
check in to the ward 5			**╳**	**╳**	**╳**			**╳**	**╳**	**╳**					
check out of the ward 6						**╳**					**╳**				**╳**
Anti-FSH Antibody Test 7			**╳**					**╳**					**╳**		**╳**
telephone follow-up 8														**╳**	
Adverse Event/Concomitant Medication Record 9		**╳**	**╳**	**╳**	**╳**	**╳**	**╳**	**╳**	**╳**	**╳**	**╳**	**╳**	**╳**	**╳**	**╳**

1. Participants received an intramuscular injection of Decapeptyl® 3.75 mg once during the 15th to 21st day of their menstrual cycle.

2. Blood FSH testing was performed 14–21 days after downregulation to determine whether the downregulation standard (FSH ≤4 IU/L) was met; if the standard was not met, retesting could be extended by up to 4 days. Blood FSH and E2 tests were repeated on D8 to 10.

3. Participants were administered the test or reference formulation on the morning of D1 and crossed over on D11.

4. PK blood sampling points in both cycles were set at D0, within 1 h before administration, and 0.5, 1, 3, 6, 9, 12, 16, 24, 32, 40, 48, 72, 96, 120, 144, and 168 h after administration, totaling 18 sampling points. At each sampling time point, 3 mL of venous blood was collected, processed according to the protocol-specified conditions and procedures, and tested for FSH. The results were used for PK parameter calculations.

5. Participants were admitted to the Phase I ward the evening before administration (D0 for the first cycle and D10 for the second cycle).

6. Participants could be discharged from the hospital after completing 48-h PK blood sample collection and safety checks on the morning of the third day after FSH administration (D3 for the first cycle and D13 for the second cycle), provided no clinically significant abnormalities were found and with the investigator’s approval.

7. Venous blood was collected for anti-FSH antibody (ADA) testing at randomization on D0, D8, D18, and upon early withdrawal from the trial.

8. During the follow-up period, the investigator conducted at least monthly telephone follow-ups to inquire about and record the subjects’ menstrual cycle recovery status. Telephone follow-ups were continued until the participants experienced two menstrual periods. For participants with regular menstrual cycles, the follow-up period for menstrual cycle recovery was three cycles from the start of downregulation; for participants with irregular menstrual cycles after withdrawal, a 6-month follow-up was conducted from the time of withdrawal.

9. From the signing of the informed consent form until the end of the trial, the occurrence of adverse events (AEs) and concomitant medication use were continuously recorded.

### 2.3 Sample collection and analysis

Blood sampling for PK analysis was conducted at the following time points: pre-dose (1 day prior and within 1 h before dosing), and 0.5, 1, 3, 6, 9, 12, 16, 24, 32, 40, 48, 72, 96, 120, 144, and 168 h post-dose, totaling 18 time points. Anti-drug antibody (ADA) samples were obtained pre-dose on Day 0, at 168 h post-dose in the first period (Day 8), at 168 h post-dose in the second period (Day 18), and at the end of the trial. At each time point, 3 mL of venous blood was collected in a clot-activator tube. If a venous indwelling needle was used, approximately 0.5 mL of blood was discarded prior to sampling to avoid contamination, and the catheter was flushed with saline after each collection. Blood samples were left to stand upright for 30 min, then centrifuged at 2,500 g for 10 min at 4 °C. The separated serum was stored at −90 °C ∼ -60 °C in ultra-low temperature freezers until PK analysis.

The serum concentration of FSH was determined using a validated electrochemiluminescence method based on the Meso Scale Discovery (MSD) platform, with two analytical linear ranges: 160 pg/mL to 16,200 pg/mL and 134 pg/mL to 4,710 pg/mL.

### 2.4 PK analysis

PK parameters were analyzed using Phoenix™ WinNonlin® software (version 8.3) with a non-compartmental analysis model. The primary PK parameters included C_max_, AUC_0-t_, and ${AUC}_{0-\infty }$ . For baseline correction of PK parameters, the arithmetic mean of the two pre-dose measurements (1 day before dosing and within 1 h before dosing) was used as the baseline. The correction process was as follows: the 0-h value in the first period was set to zero, and the post-dose values at each time point were adjusted by subtracting the baseline. If the corrected result was negative, it was set to zero.

### 2.5 Safety assessment

Safety was evaluated throughout the trial, including spontaneously reported or directly observed (AEs, abnormalities in vital signs, physical examinations, and 12-lead electrocardiograms, and clinically significant laboratory test abnormalities. ADA testing was also included in the safety assessment.

### 2.6 Sample size and statistical analysis

According to the EMA guidelines on nonclinical and clinical development of rhFSH-containing biosimilars, healthy female participants should be chosen for clinical PK comparison studies, and the sample size should ensure sufficient statistical power for bioequivalence evaluation. Considering the geometric mean ratio (GMR) of the test and reference formulations was 1.03, the estimated within-subject coefficient of variation (CV) was 30%, and the power (1-β) was 90%, the required sample size was calculated as 44 participants using a 2 × 2 crossover design. Assuming a 10% dropout rate during the trial, 48 participants were enrolled.

Bioequivalence evaluation was performed using SAS® 9.4 software. Statistical analyses were conducted on the PK parameters AUC_0-t_, ${AUC}_{0-\infty }$ , and C_max_, with a significance level of 5%. AUC_0-t_, ${AUC}_{0-\infty }$ , and C_max_ were log-transformed before mixed-effects ANOVA. The model included sequence, drug, and period as fixed effects, and participant (nested within treatment sequence) as a random effect. Bioequivalence was evaluated using two one-sided t-tests (TOST) and a (1-2α) confidence interval.

If the 90% confidence intervals of the GMRs of the PK parameters (AUC_0-t_, ${AUC}_{0-\infty }$ , and C_max_) of the test and reference formulations fall within the equivalence range of 80%–125% (including boundary values), the test and reference formulations are considered equivalent.

FSH is an endogenous substance that plays important physiological roles in the body. Its secretion varies cyclically and is influenced by circadian rhythms and individual differences. The primary objective of this study was to compare the PK parameters and evaluate the bioequivalence between the test and reference formulations. Therefore, adjustments or removal of non-drug factors that could affect the bioequivalence evaluation were made. This trial employed downregulation treatment using a GnRH-a, as described in Section 2.2, to suppress endogenous FSH levels and establish a stable baseline for bioequivalence evaluation.

Sensitivity analysis was used to assess the sensitivity of the results to certain parameter changes. PK parameters and bioequivalence statistical analyses were performed on uncorrected FSH concentrations as a sensitivity analysis to explore the impact of baseline FSH on the results.

## 3 Results

### 3.1 Participants’ demographics and baseline characteristics

**FIGURE 1:**
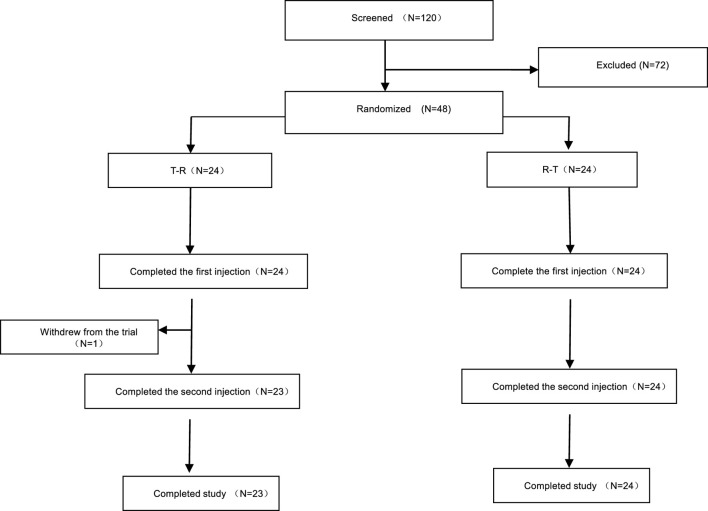
Screening and enrollment overview.

A total of 120 participants were screened, of whom 72 were excluded, and 48 were enrolled and randomly assigned to treatment groups. Group A (T-R sequence) and Group B (R-T sequence) each enrolled 24 participants. All participants in Group B completed the trial, whereas one participant (R038) in Group A withdrew early, and the remaining 23 participants in Group A completed the trial. Details are shown in Figure 1. Demographic and baseline characteristics are summarized in Table 2 and were well balanced between the two groups.

**TABLE 2:** participants’ demographics and baseline characteristics by treatment sequence.

Sequence	Group T-R (n = 24)	Group R-T (n = 24)	p-value (t-test)
Age, years			
Mean ± SD	25.54 ± 4.74	28.13 ± 5.65	0.093
Min–max	20,36	21,39	
Ethnicity, N, %			
Han	19 (79.2%)	21 (87.5%)	
Others	5 (20.8%)	3 (12. 5%)	
Height, cm			
Mean ± SD	158.93 ± 5.164	156.46 ± 4.863	0.095
Min–max	150.2, 173.5	145.9,164.0	
Weight, k			
Mean ± SD	55.60 ± 6.642	54.57 ± 7. 167	0.605
Min–max	46.5,69.5	45.4,71.6	
BMI, kg/m^2^			
Mean ± SD	22. 00 ± 2.108	22.3 0 ± 2 0.752	0.669
Min–max	18.8, 26.0	18.5, 27.9	

### 3.2 PK properties

**FIGURE 2:**
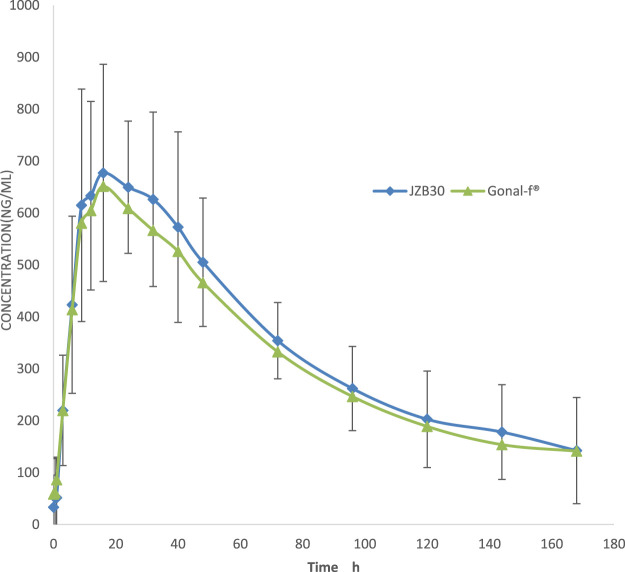
Serum concentration–time curves. The mean (±SD) serum concentration–time curves of JZB30 and Gonal-f®.

Following administration, the mean serum concentrations of the test and reference formulations gradually increased, reached a peak, and then slowly declined, demonstrating comparable PK profiles (Figure 2). PK parameters included C_max_, AUC_0-t_, ${AUC}_{0-\infty }$ , T_max_, t_1/2_, and λz, %AUC_ex_. The PK parameter results are presented in Tables 3, 4. Analysis of variance (ANOVA) of the primary PK parameters for FSH (C_max_, AUC_0-t_, ${AUC}_{0-\infty }$ ) revealed that period and formulation significantly influenced Cmax and AUC_0-t_ (P < 0.05), while period also had a significant effect on AUC ${AUC}_{0-\infty }$ (P < 0.05); however, sequence showed no significant impact on Cmax or AUC_0-t_ (P > 0.05), and neither sequence nor formulation significantly affected ${AUC}_{0-\infty }$ (P > 0.05). Complete ANOVA results for these key PK parameters are presented in Tables 5, 6.

**TABLE 3:** PK parameters of recombinant human follicle-stimulating hormone injection.

PK parameter (Units)	Mean ± SD (CV%)	
	Test formulation (N = 47)	Reference formulation (N = 47)
Cmax (pg/mL)	842 ± 265 (31.5%)	801 ± 250 (31.3%)
AUC0-t (h*pg/mL)	7.41 × 10^4^ ± 1.76 × 10^4^ (23.8%)	7.02 × 10^4^ ± 1.62 × 10^4^ (23.1%)
AUC 0−∞ (h*pg/mL)	1.33 × 10^5^ ± 6.99 × 10^4^ (52.7%)	1.15 × 10^5^ ± 4.41 × 10^4^ (38.5%)
%AUCex	36.5 ± 16.9 (46.3%)	35.1 ± 12.5 (35.7%)
Tmax(h)	16.0 (9.00, 40.0)	24.0 (6.00, 32.0)
λz (1/h)	0.0074 ± 0.0040 (54.1%)	0.0074 ± 0.0029 (39.2%)
t1/2(h)	56.0 ± 17.9 (32.0%)	51.2 ± 0.43 (0.8%)
CL/F (mL/h)	152 ± 63.1 (41.5%)	161 ± 50.4 (31.3%)
Vd/F (mL)	2.32 × 10^4^ ± 7.82 × 10^3^ (33.7%)	2.36 × 10^4^ ± 6.98 × 10^3^ (29.6%)

Cmax :maximum concentration; AUC0-t: area under the concentration-time curve from time 0 to the time of last observed concentration; AUC 0−∞ : area under the concentration-time curve from time 0 to infinity; %AUCex:percentage of AUC extrapolated, calculated as ( ${AUC}_{0-\infty }$ – AUC_0-t_)/ ${AUC}_{0-\infty }$ × 100%; Tmax: time at which Cmax occurred; λz:terminal elimination rate constant, obtained by log-linear regression of the terminal plasma concentration-time profile; t1/2 apparent half-life of terminal elimination; CL/F: apparent oral clearance; Vd/F: apparent volume of distribution.

Tmax is presented as median (minimum, maximum).

If %AUCex >20%, t_1/2_ is not included in the summary.

**TABLE 4:** PK parameters of recombinant human follicle-stimulating hormone injection after baseline correction.

PK parameter	Mean ± SD (CV%)	
	Test formulation (N = 47)	Reference formulation (N = 47)
Cmax (pg/mL)	748 ± 234 (31.3%)	707 ± 231 (32.6%)
AUC_0_-t (h·pg/mL)	5.82 × 10^4^ ± 1.26 × 10^4^ (21.6%)	5.44 × 10^4^ ± 1.39 × 10^4^ (25.5%)
${AUC}_{0-\infty }$ (h·pg/mL)	9.28 × 10^4^ ± 6.09 × 10^4^ (65.6%)	7.70 × 10^4^ ± 3.16 × 10^4^ (41.0%)
%AUCex	27.2 ± 18.7 (68.8%)	23.7 ± 16.3 (68.8%)
Tmax (h)	16.0 (9.00, 40.0)	24.0 (6.00, 32.0)
λz (1/h)	0.0112 ± 0.0076 (67.9%)	0.0147 ± 0.0141 (96.0%)
t_1_/_2_ (h)	45.7 ± 15.3 (33.5%)	33.7 ± 14.8 (43.9%)
CL/F (mL/h)	222 ± 95.0 (42.8%)	262 ± 144 (55.0%)
Vd/F (mL)	2.45 × 10^4^ ± 9.71 × 10^3^ (39.6%)	2.28 × 10^4^ ± 8.13 × 10^3^ (35.7%)

**TABLE 5:** ANOVA results for major PK parameters of recombinant human follicle stimulating hormone injection.

PK parameter	n(T)	n(R)	P-value		
			Sequence	Period^#^	Formulation
C_max_	47	47	0.205	0.001	0.027
AUC_0-t_	47	47	0.375	0.000	0.009
${AUC}_{0-\infty }$	47	47	0.821	0.000	0.051

**TABLE 6:** ANOVA results for baseline-corrected major PK parameters of recombinant human follicle stimulating hormone injection.

PK parameter	n(T)	n(R)	P-value		
			Sequence	Period^a^	Formulation
C_max_	47	47	0.049	0.000	0.017
AUC_0-t_	47	47	0.019	0.000	0.001
${AUC}_{0-\infty }$	47	47	0.116	0.000	0.004

a “Period” refers to the statistical period effect in the crossover design, i.e., the difference between Period 1 and Period 2, regardless of the treatment sequence.

### 3.3 Bioequivalence assessment

Based on the bioequivalence analysis set (BES, N = 47), the primary PK parameters (C_max_, AUC_0-t_, ${AUC}_{0-\infty }$ ) were evaluated for bioequivalence. The results are shown in Table 5.

The GMR of Cmax was 106.12%; the GMR of AUC0-t was 108.04%; and the GMR of AUC 0−∞ was 115.32%. Using the 90% confidence intervals of the GMRs of the test and reference formulations, all parameters (Cmax, AUC0-t, ${AUC}_{0-\infty }$ ) met the criteria for bioequivalence.

Sensitivity analysis was performed on uncorrected FSH concentrations, and the results are shown in Table 7. The sensitivity analysis results were consistent with the main analysis results, indicating the robustness of the bioequivalence conclusion.

**TABLE 7:** Bioequivalence assessment of recombinant human follicle stimulating hormone injection.

PK parameter	Least squares geometric Mean (GeoLSmean)				Within-subject variability (%)
	T (n = 47)	R (n = 47)	T/R (%)	90%CI (%)	
C_max_ ^a^	717	675	106.1	101.9, 110.5	11.7
C_max_ ^b^	809	770	105.1	101.3, 109.0	10.6
AUC_0-t_ ^a^	5.68 × 10^4^	5.26 × 10^4^	108.0	104.1, 112.1	10.7
AUC_0-t_ ^b^	7.20 × 10^4^	6.86 × 10^4^	105.1	101.9, 108.3	8.8
${AUC}_{0-\infty }$ ^a^	8.16 × 10^4^	7.07 × 10^4^	115.3	106.5, 124.9	23.3
${AUC}_{0-\infty }$ ^b^	1.19 × 10^5^	1.08 × 10^5^	109.9	101.6, 119.0	23.2

a Baseline-Corrected

b Uncorrected.

### 3.4 Safety assessment

Safety analysis was conducted only on adverse events occurring after the administration of the test or reference formulations, in accordance with the protocol. Therefore, the safety analysis population included 48 participants who received the test or reference formulations, and safety events in four participants who only received Decapeptyl were reported separately. All adverse events observed during the trial were systematically assessed for causality in relation to the investigational products (JZB30 or Gonal-f®). This causality assessment was conducted by the investigators based on temporal relationship, known pharmacological effects of rhFSH, and clinical judgment. Some adverse events were assessed as “unrelated” to the study drugs. These included common non-specific events such as High Blood Triglyceride levels and elevated blood creatine phosphokinase. In contrast, events related to the reproductive system (e.g., vaginal bleeding) were assessed as “possibly related” to the study drugs. This classification was based on the known pharmacological action of FSH in stimulating ovarian activity. It is important to emphasize that even these “possibly related” events were mild (Grade 1), transient, and resolved spontaneously without any medical intervention.

Safety analysis results showed that 21 participants experienced 27 adverse events, with an overall adverse event incidence of 43.8%. Among those receiving the test formulation, 12 participants experienced 15 adverse events, with an adverse event incidence of 25.0% and an adverse reaction incidence of 20.8%. Among those receiving the reference formulation, 12 participants experienced 12 adverse events, with an adverse event incidence of 25.5% and an adverse reaction incidence of 17.0%. The incidence and severity of adverse events were comparable between the test and reference formulations.

No adverse events led to withdrawal, no serious adverse events occurred, and no adverse events were graded as three or higher. Adverse events by system organ class and preferred term for T and R are presented in Table 8

**TABLE 8:** Summary of TEAEs by system organ class and preferred term (SS).

System organ class Preferred term	T (N = 48) n (%)E	R (N = 47) n (%)E	Total (N = 48) n (%)E
At least one TEAE	12 (25.0) 15	12 (25.5) 12	21 (43.8) 27
Reproductive System and Breast Disorders	8 (16.7) 8	6 (12.8) 6	14 (29.2) 14
Vaginal Bleeding	7 (14.6) 7	6 (12.8) 6	13 (27.1) 13
Ovarian Cyst	1 (2.1) 1	0 (0.0) 0	1 (2.1) 1
Various Investigations	3 (6.3) 5	4 (8.5) 4	7 (14.6) 9
Leukopenia	3 (6.3) 3	1 (2.1) 1	4 (8.3) 4
Low Neutrophil Count	2 (4.2) 2	0 (0.0) 0	2 (4.2) 2
Positive Antibody Test	0 (0.0) 0	1 (2.1) 1	1 (2.1) 1
High Blood Triglyceride levels	0 (0.0) 0	1 (2.1) 1	1 (2.1) 1
Elevated Creatine Phosphokinase	0 (0.0) 0	1 (2.1) 1	1 (2.1) 1
Vascular and Lymphatic System Diseases	0 (0.0) 0	2 (4.3) 2	2 (4.2) 2
Hot flush	0 (0.0) 0	2 (4.3) 2	2 (4.2) 2
Various Neurological Diseases	1 (2.1) 1	0 (0.0) 0	1 (2.1) 1
Dizziness	1 (2.1) 1	0 (0.0) 0	1 (2.1) 1
Gastrointestinal System Diseases	1 (2.1) 1	0 (0.0) 0	1 (2.1) 1
Abdominal Pain	1 (2.1) 1	0 (0.0) 0	1 (2.1) 1

T: test formulation; R: reference formulation; n: Number of subjects experiencing at least one adverse event; %: Percentage of subjects experiencing at least one adverse event; E: number of adverse events; Adverse events coded using MedDRA, 24.1.

### 3.5 Immunogenicity assessment

48 participants were enrolled in the trial. One participant (R038, T-R) withdrew from the study 6 h after the first dose, having only provided a baseline ADA sample. The remaining 47 participants completed the two dosing periods and provided ADA samples at baseline, Day 8, and Day 18.

One participant (R044, T-R) tested positive for anti-FSH antibodies at Day 18. No anti-FSH antibodies were detected after administration of the test formulation, and only one participant [1/47] tested positive after receiving the reference formulation. The antibody response turned negative after approximately 6 months without any intervention. These results indicate that the drug exhibited low immunogenicity in the majority of participants.

## 4 Discussion

### 4.1 FSH endogenous characteristics

FSH is an endogenously produced substance. In the bioequivalence studies of Gonal-f® biosimilars Ovaleap® and Bemfola®, gonadotropin-releasing hormone agonists (GnRH-a) were used to downregulate endogenous FSH to prevent individual variations in endogenous FSH secretion from affecting the bioequivalence evaluation. GnRH-a acts by continuously stimulating GnRH receptors, leading to receptor downregulation and subsequent suppression of pituitary gonadotropin secretion, including FSH and LH. This mechanism effectively reduces endogenous hormone levels, providing a stable baseline for PK assessment.

According to the “Expert Consensus on Ovulation Induction Drugs in Assisted Reproduction” (Le Contonnec et al., 1994) published by the Chinese Society of Reproductive Medicine in 2015, GnRH-a can be used to downregulate FSH and LH in controlled ovarian stimulation (COS) protocols. After 7–14 days of GnRH-a administration, drug-induced pituitary-ovarian suppression is achieved, reducing endogenous FSH and LH secretion to low levels. The 14-day time point was chosen for downregulation evaluation because it corresponds to the period when GnRH-a typically reaches its maximal suppressive effect. In this study, 3.75 mg of triptorelin acetate for injection (Decapeptyl®) was administered 14 days before the first dose to achieve downregulation. On Day 0, participants were evaluated for downregulation, and those meeting the requirement (FSH ≤4 IU/L) proceeded to the subsequent dosing of the test and reference formulations. The threshold of FSH≤ 4 IU/L, which has been applied in previous pivotal bioequivalence studies of rhFSH biosimilars (Lammerich et al., 2015) (Lugan et al., 2005) indicates effective suppression of endogenous FSH, minimizing individual hormonal fluctuations that could confound PK and bioequivalence evaluations.

### 4.2 Dose selection rationality

According to the PK study of Ovaleap®, the linear PK range for a single-dose subcutaneous injection of rhFSH spans at least from 37.5 to 300 IU. The “Expert Consensus on Ovulation Induction Drugs in Assisted Reproduction” published by the Chinese Society of Reproductive Medicine in 2015 recommends that the maximum daily dose of FSH in ovulation induction (OI) protocols should not exceed 225 IU, while the initial dose range in COS protocols is 112.5–300 IU/day. According to the EMA guidelines for the nonclinical and clinical development of rhFSH-containing biosimilars, a dose within the linear portion of the dose-response curve should be selected to sensitively detect potential differences. In our study, PK parameters were used as endpoints to evaluate the bioequivalence between the test and reference formulations, with the linear PK range serving as a partial surrogate for the linear dose-response range. Considering the product specification of 5.5 μg (75 IU) and the clinical dosing practices, including the maximum dose used in China, a dose of 225 IU was chosen for this study. Therefore, a single subcutaneous injection of 225 IU was administered as the dosing regimen.

### 4.3 Data handling method and its impact

In bioequivalence studies, the choice of data handling methods is crucial for the accuracy and reliability of the results (Dis and sanayake, 2010). FSH is an endogenous hormone whose circulating levels exhibit natural fluctuations influenced by the menstrual cycle, circadian rhythms, and individual physiological differences. Although downregulation was successful (FSH ≤4 IU/L), low levels of endogenous FSH persist and demonstrate minor intra- and inter-individual variations. Baseline correction is performed precisely to isolate the PK signal attributable solely to the injected drug from this residual background “noise.” Baseline correction was performed for the bioequivalence (BE) studies of the Gonal-f® biosimilars Ovaleap® and Bemfola®. Although baseline correction can improve statistical power, in some cases, uncorrected data analysis may already provide reliable conclusions. When correction methods are complex or not applicable, using uncorrected data may be more reasonable. In this study, baseline-corrected data were used for evaluation, and sensitivity analysis was performed using uncorrected data to assess the impact of baseline correction on the study results. This approach allowed a comprehensive assessment of the similarity between the biosimilar and the reference drug, leading to more robust conclusions.

Variance analysis was conducted on sequence, period, and formulation factors to evaluate their impact on the primary PK parameters (C_max_, AUC_0-t_). Before baseline correction, period and formulation had statistically significant effects on C_max_ and AUC_0-t_ (P < 0.05), indicating these factors significantly affected the PK parameters without correction. The formal statistical test for carryover effects, manifested as the sequence effect in the ANOVA, demonstrated no significant sequence effect for any primary PK parameter in the uncorrected data (all P > 0.05, Table 5). This provides key statistical evidence against the presence of a clinically meaningful carryover effect. Although the sequence factor reached nominal significance for some parameters after baseline correction, this is more likely attributable to a chance imbalance in baseline FSH levels between the sequence groups, an difference that was accentuated by the correction procedure itself. This phenomenon may be related to natural fluctuations in follicle-stimulating hormone (FSH) levels, which vary over time and physiological status, especially during the menstrual cycle in women. Therefore, when designing the dosing sequence, it is essential to consider these natural fluctuations to minimize their impact on trial results. The inclusion criteria for this study specified a menstrual cycle length of 25–34 days (inclusive) and scheduled downregulation between days 15 and 21 of the menstrual cycle. This design aimed to standardize the downregulation process and reduce interference from natural fluctuations in endogenous FSH levels on the trial outcomes.

### 4.4 Immunogenicity assessment

Immunogenicity assessment in this study revealed a single, transient anti-FSH antibody positive case following administration of the reference product, which reverted to negative spontaneously during the approximately 6-month follow-up period without associated PK alterations or clinical adverse events. This finding aligns with the well-documented low immunogenicity profile of marketed rhFSH products, largely attributable to their high structural homology with endogenous FSH.

Although the theoretical risk exists that persistent neutralizing antibodies could impact the bioactivity and efficacy of exogenous FSH, accumulated clinical evidence suggests that antibody events associated with rhFSH are predominantly transient and non-neutralizing. The isolated event in our study did not impact the bioequivalence conclusion, underscoring its likely limited clinical relevance. Considering the demonstrated PK bioequivalence, comparable safety profile, and this self-limiting immunogenicity event between JZB30 and the reference product in healthy women, JZB30 exhibits a similarly low immunogenicity risk profile.

## 5 Conclusion

This study demonstrates that JZB30 is bioequivalent to Gonal-f® in healthy adult Chinese female participants, with a comparable safety profile and a low incidence of immunogenicity in the short term. It is important to note that the immunogenicity data were derived from a Phase I study with limited dosing and observation period. The final confirmation of the immunogenicity profile of JZB30 will require evaluation in the target patient population undergoing multiple treatment cycles in longer-term Phase III clinical studies.These findings provide strong scientific support for the clinical application of JZB30 as a biosimilar to Gonal-f®.

## Data Availability

The raw data supporting the conclusions of this article will be made available by the authors, without undue reservation.
